# Career satisfaction of German human genetics residents

**DOI:** 10.1515/medgen-2021-2103

**Published:** 2022-01-12

**Authors:** Johanna Tecklenburg, Robert Meyer, Ilona Krey, Brigitte Schlegelberger

**Affiliations:** Institut für Humangenetik, Medizinische Hochschule Hannover, Carl-Neuberg-Straße 1, 30625 Hannover, Germany; Institut für Humangenetik, Universitätsklinikum Aachen, Pauwelsstraße 30, 52074 Aachen, Germany; Institut für Humangenetik, Universitätsklinikum Leipzig, Philipp-Rosenthal-Str. 55, 04103 Leipzig, Germany

**Keywords:** specialist training, residents, career satisfaction, future of human genetics

## Abstract

**Objectives:**

The aim of this survey was to investigate the career satisfaction of human genetics residents in Germany and to analyse the influence of intrinsic and extrinsic factors.

**Methods:**

We developed an online survey for the evaluation of a broad range of factors concerning the situation of human genetics residents in Germany using validated questionnaires and adding human genetics specific items to them. Human genetics residents working at institutions with an authorization for specialist training were asked to participate in the online survey. To analyse the situation of specialist training in human genetics and the influence of multiple factors on career satisfaction, descriptive statistics, mean descriptive statistics and comparisons of mean values as well as multiple linear regression analyses were carried out.

**Results:**

Of the 71 institutions contacted, 41 (58 %) provided feedback and reported the number of 114 residents in human genetics. In total, 58 residents completed the questionnaire (50.9 %). Overall career satisfaction was high with a mean score of 30.8 (scale ranging from 8–40). Factors significantly influencing career satisfaction were general life satisfaction, occupational self-efficacy expectations and content with the doctors entitled to the specialty training. Except for the reduced perception to achieve their professional goals expressed by women with children, career satisfaction was influenced by neither gender nor parental status, other sociodemographic factors, variables concerning the personal professional life and the residency in general, the subjective perceived workload nor the site of specialist training. Participation in research activities differed significantly between male and female residents. The residents’ assessment of their own professional prospects and the prospects of the subject were consistently positive, even though residents consider the current requirement planning by the GB-A for human geneticists as inappropriate and believe that human genetics is not yet firmly anchored as a specialist discipline in the consciousness of other medical colleagues and the general public.

**Conclusions:**

Career satisfaction of German human genetics residents is generally high and mainly influenced by life satisfaction, occupational self-efficacy expectations and quality of the specialist training. In contrast to other specialties career satisfaction seems to be independent from gender or parental status even though male residents were significantly more often involved in research activities. In order to keep human genetics residents in the specialty, measures that enable balanced professional and care work as well as continuous improvement of specialist education, e. g. through the implementation of structured curricula and continuing education of the doctors entitled to specialist training, is of great importance.

## Introduction

Physician shortages in Germany, an issue that has been particularly challenging for rural areas and small disciplines, have become an increasing concern in health care as well as among health scholars and policy makers in the last years. In this context a focus was placed on the career satisfaction of residents and the conditions of specialty training reflecting the increasing importance of the resource “clinician.” In 2009 the German Medical Association first conducted a survey among German residents to determine the quality of specialist training in Germany assuming a correlation between the quality of specialist training and motivation for leaving medical care and moving into other professions. To ensure comprehensive and high-quality patient care, not only a sufficient number of medical graduates, but also their satisfaction in the course of their careers is important [1]. So far, specific data on the subjective assessment of specialist training in human genetics have not been available. We therefore conducted a survey to obtain data on the quality of specialist training and on factors influencing career satisfaction and life satisfaction among human genetics residents in Germany.

## Methods

For the development of our questionnaire, we adapted validated questionnaires from the literature described below. In expert rounds with three members of the “Junge Humangenetik,” the forum for young doctors and scientists of the German professional society for human genetics (GfH e. V.), further items were added to the questionnaire regarding sociodemographic variables, variables concerning the institution of residency training and variables concerning the personal professional life and residency in general. The complete questionnaire can be obtained from the corresponding author. After an initial pre-test stage 59 items were chosen for the final questionnaire. In addition, the submission of free text answers was available for certain topics. The online questionnaire was realized using SoSci Survey (Leiner, D. J. (2019). SoSci Survey (Version 3.1.06) [computer software]; available at https://www.soscisurvey.de) and made available to participants at https://webext.mh-hannover.de/soscisurvey/humangenetik2020/. Data were collected anonymously and stored on a server of the Hannover Medical School.

By using the publicly available databases of the 17 state medical associations in Germany we identified 71 institutions employing doctors who are entitled to further specialist training. In each institution one physician (to avoid multiple feedback) was sent an e-mail and informed about the planned survey and asked to provide feedback on whether and how many residents were employed in the institution. In a second step those institutions who had provided feedback were sent the link for the online questionnaire and asked to redirect it to their residents. In addition, we used the mailing list of the “Junge Humangenetik” to distribute the link for the survey. The survey was available for 10 weeks from January to March 2021. Two reminder e-mails were sent.

### Data recruitment

For the variable “career satisfaction” a score modified after Römer et al. [1] was calculated from eight items. For the items a five point Likert scale was used ranging from 1–5 (“strongly disagree” = 1, “strongly agree” = 5). Overall, scores between 8 and 40 points could be achieved.

Further independent variables influencing career satisfaction were obtained:

– Socio-demographic variables.
– Variables concerning the institution of residency training.
– Variables concerning the personal professional life and the residency in general.
– Intrinsic variables:
  – Measurement of occupational self-efficacy expectations, a six item scale that measures aspects of motivation and ability as validated by Abele et al. [2] The score ranges from 6–30.
  – For the dependent variable “life satisfaction” the Questions on Life Satisfaction (FLZM) as validated by Herschbach et al. [3] were used. The general FLZM measures the participants’ subjective importance of and satisfaction with eight areas of life, resulting in a weighted life satisfaction with scores ranging from −96 to 160.
  – For the measurement of the subjective perceived workload a modified version of the “National Aeronautics and Space Administration-Task Load Index” (NASA-TLX) as validated by Flägel et al. for measuring workload in general practice was used [4]. Participants were asked to evaluate their last 10 consultations on a five point Likert scale ranging from 1–5 (“not/nothing at all” = 1, “very much/a lot” = 5) in terms of mental demand, time pressure, frustration, effort and satisfaction with own performance. Overall scores between 5 and 25 could be achieved.
  – For the subjective evaluation of the residency institution and the doctors responsible for the specialty training, a modified version of the validated questionnaire by Prien et al. [5] with a five point Likert scale ranging from 1–5 (“strongly disagree” = 1, “strongly agree” = 5) for 11 and 18 items, respectively, was used. Scores between 11–55 and 18–90 could be achieved, respectively, and a higher score reflected a good overall rating.

Participants were furthermore asked to rate statements about the future of the discipline and their own professional future on a five point Likert scale ranging from 1–5 (“strongly disagree” = 1, “strongly agree” = 5).

### Statistical analysis

The statistical analyses were carried out with GraphPad Prism version 9.2.0 for Windows, GraphPad Software, San Diego, California USA (www.graphpad.com). The significance level was set to α=0.05.

The individual items on career satisfaction as well as the respective total scores (see below) were compared between male and female doctors and male and female doctors with and without children. The *t*-test for independent samples was used for this purpose.

Fisher’s exact test was used to assess differences in research activities of male and female residents.

The weighted scores on life satisfaction (see below) were compared with standard values as validated by Daig et al. [6] The *t*-test for independent samples was used for this purpose.

To determine which variables influence career satisfaction and life satisfaction a multiple linear regression was calculated. Dummy variables were created for categorical variables. In a first step we performed a simple linear regression of the dependent variables “career satisfaction” and “life satisfaction” with the surveyed independent variables.

For the dependent variable “career satisfaction” the independent variables occupational self-efficacy, life satisfaction and evaluation of the doctors responsible for the specialty training achieved a *P*-value of <0.0001 in the simple linear regression and were included in the final model of regression.

## Results

### Participants

Of the 71 institutions contacted, 41 (58 %) provided feedback on whether and how many residents they employ and reported the number of 114 residents employed in human genetics. Of those, 34 (29.8 %) were employed in private practice or medical care centres and 80 (70.2 %) at university departments. In total 58 residents completed the questionnaire (50.9 %), and of those, 20 (34.5 %) were employed in private practice or medical care centres and 38 (65.5 %) at university departments, reflecting the distribution of the 114 reported residents. Table 1 summarizes sociodemographic and academic characteristics of the participants. Although the response rate was rather high, certain items were answered by a small number of residents. Thus, these data need to be analysed with caution. Efforts should be taken to confirm the results by an independent survey, and to involve as many institutions with an authorization for specialist training as possible. Furthermore, it can be assumed that the results of this survey could be distorted by the increased participation of those who give the topic of specialist training a high priority (compare Scholz et al.[7]).

**Table 1:** Selection of sociodemographic and academic characteristics of participating residents.

Characteristics of the participating residents	*N* (%)
Age	
*25–34*	39 (67.2)
*35–44*	15 (25.8)
*≥45*	4 (7)
*Median age*	33.8 years (± 5.3)
Gender	
*Male*	20 (34.5)
*Female*	38 (65.5)
*Diverse*	0 (0)
Marital status	
*Single*	7 (12.3)
*Solid partnership*	19 (33.3)
*Married*	31 (54.4)
*Divorced*	0 (0)
*Widowed*	0 (0)
Children	
*None*	28 (49.1)
*≥1*	29 (50.9)
Years of residency	
*1*	4 (6.9)
*2*	16 (27.6)
*3*	15 (25.9)
*4*	11 (19.0)
*≥5*	12 (20.6)
Doctorate	
*Yes*	46 (79.3)
*No*	12 (20.7)
Research activities	
*Yes*	32 (55.2)
*No*	26 (44.8)

**Table 2:** Mean value comparisons of items measuring career satisfaction by gender and parental status. t-Values reflect the comparison of male/female residents without children to male/female residents with children, respectively. 1 = strongly disagree, 5 = strongly agree; score ranging from 8–40.

Item	Total cohort		Female residents w/o children		Female residents with children		t-Test	Male residents w/o children		Male residents with children		t-Test
	Mean	SD	Mean	SD	Mean	SD	*P*	Mean	SD	Mean	SD	*P*
I would study medicine again	4.0	1.1	3.6	1,4	4.5	0,7	0.0169	3,8	1,2	4,1	1	0.5512
I am satisfied with my current working hours	3.9	1.2	3.8	1.2	4.1	1.2	0.4460	4.1	1.1	3.8	1.4	0.6007
I am satisfied with my current salary	4.0	0.9	3.9	0.8	4.1	1.3	0.5715	4.1	0.9	3.7	0.7	0.2819
I would start my specialist training in human genetics again	4.4	0.8	4.7	0.6	4.3	0.7	0.0667	3.9	1.2	4.3	0.7	0.3746
So far, I have been able to achieve my personal professional goals	3.8	1.0	4.1	0.8	3.3	1.1	0.0147	4	0.8	3.9	1	0.8078
To date, my professional life has turned out the way I expected it to at the beginning	2.8	1.2	2.9	1.1	2.4	1.3	0.2088	3.6	1.1	2.6	1.3	0.0798
Close friends would say that I have made good progress in my professional life	4.0	0.9	4.3	0.7	3.5	1	0.0071	4.1	1	4	0.7	0.7985
Overall, I am satisfied with my professional development	4.0	0.8	4.2	0.9	3.7	0.9	0.0954	4.3	0.7	3.8	0.6	0.1035
Total score	30.8	4.8	31.5	4.2	29.8	5.7	0.3023	31.9	4.9	30.2	4.0	0.4065

**Table 3:** Multiple linear regression on the dependent variable “career satisfaction.” The beta coefficients can be considered to estimate the relative contribution of each predictor to the overall prediction of the dependent variable career satisfaction.

Variable	Regression coefficient B	Standard error	Beta	*P*
Intercept	5.489	3.629		
Life satisfaction	0.053	0.015	0.313	0.0009
Occupational self-efficacy expectations	0.643	0.143	0.443	<0.0001
Evaluation of the doctors entitled to the specialty training	0.093	0.038	0.250	0.0186

### Comparison of career satisfaction by gender and parental status

Overall career satisfaction was high among human genetics residents: The mean of the total score was 30.8 (on a scale from 8 to 40). The item rated highest was “I would start my specialist training in human genetics again” with a mean score of 4.4 (maximum 5), and the item rated lowest was “To date, my professional life has turned out the way I expected it to at the beginning” with a mean score of 2.8. When differentiating according to gender and parental status, male residents without children reported the highest career satisfaction with a mean score of 31.9, followed by female residents without children with a mean score of 31.5. Residents with children displayed lower levels of career satisfaction, with male residents with children reporting a mean score of 30.2 and female residents with children reporting a mean score of 29.8. However, none of the differences of total scores as well between the different groups were statistically significant. In regard to single item scores, women with children had significantly lower scores with regard to achieving their professional goals and to their perceived progress in their professional life compared to women without children (P=0.0147 and P=0.0071, respectively). In contrast, no significant differences were found between men with and men without children (Table 2).

### Institution of residency training and research activities

Of all participants, 65 % receive their specialty training at an academic institution and 35 % in private practice. This ratio is the same for male and female residents. Of all participants, 55.2 % are involved in research activities. The distribution between male and female residents varies significantly: 80 % of male residents participate in research activities, whereas only 42.1 % of female residents are involved in research (P=0.0114). This ratio remained stable when stratifying for the institution of specialty training and for parental status. In academic institutions 92.3 % of male residents participate in research compared to 60 % of female residents. In private practice 57.1 % of male and 7.7 % of female residents participate in research. When differentiating by parental status, 77.8 % of male residents with children participate in research compared to 41.2 % of female residents with children. In our survey, although significant, this difference did not influence career satisfaction.

### Subjective perceived workload

In our modified version of the NASA-TLX five items were asked reflecting the necessary effort, pressure and self-assessed performance for the genetic counselling carried out by residents. A maximum score of 5 for each item and a total score of 25 could be achieved, where a higher score reflected a higher degree of stress. A mean score of 12.9 (± 3.0) was measured among the survey participants. The highest average was observed for the item “mental demand” (mean 3.4 ± 1.0), the lowest for “frustration” (mean 1.8 ± 1.2). The feeling of time pressure was rated with a mean score of 2.7 (± 1.2), the general effort in the consultation was rated with a mean score of 2.9 (± 0.9) and satisfaction with the own performance was rated with a mean score of 3.9 (± 0.6).

### Influence on career satisfaction

The independent variables “life satisfaction,” “occupational self-efficacy expectations” and “evaluation of the doctors responsible for the specialty training” explained 53.5 % of the variance of the dependent variable “career satisfaction” (adjusted R^2^ 0.5345); the F-test was significant (F = 22.82, P<0.0001). Table 3 shows the results of the multiple linear regression.

Interestingly, neither sociodemographic factors (including gender, parental status and age), variables concerning the institution of residency training (such as type of institution, presence of a mentor, dealing with overtime and support in achieving the necessary numbers and content for specialty training), variables concerning the personal professional life and the residency in general (such as time spent in another specialty, completed doctorate, research activities and working part-time) and subjective perceived workload nor the evaluation of the residency institution significantly influenced career satisfaction of human genetics residents.

### Occupational self-efficacy expectations and life satisfaction

In this model the highest influence on career satisfaction came from the occupational self-efficacy expectations (beta = 0.443; B = 0.642; P<0.0001). On the six item questionnaire measuring aspects of motivation and abilities a maximum total score of 30 could be achieved. The mean score was 23.9 (± 3.2) (minimum 15; maximum 30). There was no statistically significant correlation between occupational self-efficacy and subjective perceived workload.

The second largest influence came from adjusted life satisfaction (beta = 0.443; B = 0.053; P=0.0009). Since life satisfaction significantly influenced career satisfaction, a closer look was taken at the life satisfaction in the cohort of human genetics residents and this was compared to standard values obtained by Daig et al. [6] In the total cohort, life satisfaction was lower compared to standard values in all age groups. Female residents showed lower life satisfaction than control groups in all ages groups as well as male residents aged 35–44 and 45–54, whereas male residents aged 25–34 displayed a higher life satisfaction than controls. However, none of these differences was statistically significant except for the small group of female residents in the age group of 35–44 (33.22 ± 29.34 vs. 71.39 ± 38.35; P=0.003; n=9, not representative).

### Evaluation of the doctors entitled to the specialty training

In general, satisfaction with the doctors entitled to the specialty training was high. With a possible total score ranging from 18–90 the mean score was 73 (± 12.3) (minimum 43; maximum 90). The quality of the specialty training significantly influenced career satisfaction (beta = 0.250, B = 0.093; P=0.0186).

### Opinion on the future of human genetics in Germany

The residents’ assessment of their own professional prospects and the prospects of the subject were consistently positive. Of all participants, 93.1 % believe that the importance of the subject will increase in the future and 74.1 % rate their own career opportunities as good (rating of 4 or 5 on the five point Likert scale). However, only 3.4 % think that human genetics is firmly anchored as a specialist discipline in the consciousness of other human colleagues and none of the participants of the survey agreed with the statement that human genetics is firmly anchored as a specialist discipline in the awareness of the general population. Moreover, 5.2 % believe that the current requirement planning of the Joint Federal Committee (Gemeinsamer Bundesausschuss) of one specialist per 600,000 inhabitants is realistic or appropriate.

## Discussion

Recent years have brought a substantial increase in genetic knowledge that goes hand in hand with an increasing demand for genetic services and genetic counselling. The number of specialist doctors for human genetics in Germany is currently around 360 [7]. The need for genetic counselling in Germany can be estimated at some 100,000 consultations per year, considering that more than 25,000 patients per year are newly diagnosed with a hereditary cancer, around 47,000 children per year are born with a genetic condition or a malformation, more than 3 million people are affected by a rare genetic disease and 8,500 invasive prenatal examinations are carried out each year, with a subsequent need for genetic counselling and the resulting need of care for family members at risk [8]. The estimated current need for care can already hardly be met with the existing number of specialists today. Due to advantages in genetic knowledge and the increase in therapeutic options, the need for genetic services and counselling will increase dramatically in the next years, which makes it even more important to regard residents as a valuable resource that will ensure human genetics care in Germany in the future. A cornerstone for securing a high standard of specialist care in human genetics is a high-quality specialist training. However, satisfaction with one’s own career is at least as important in order to prevent migration to other disciplines or to other professions.

As early as 1989 Schwartz et al. identified a “controllable lifestyle” (CL) as a new factor in career choice by medical students, for both women and men. Specialties featuring a CL, i. e. control of work hours, work load and work–life balance, included, among others, anaesthesiology, pathology and radiology, whereas specialties like surgery, internal medicine, paediatrics or obstetrics–gynaecology were defined as non-CL specialties. It was observed that the percentage of top medical students in the USA (i. e. those who were able to obtain the specialty of their choice) entering CL specialties had significantly increased over the last decade [9]. This trend has been confirmed in recent years, also outside the USA. Residents place more emphasis on work–life balance when selecting their specialty and prefer CL specialties over non-CL specialties. Furthermore, residents are more likely to change their choice of specialty, contrary to their professional interest, because of social reasons [10]. The increasing feminization of medicine requires innovative and flexible working time models and part-time career options that enable shared care work in the family. In this context human genetics can be regarded as a CL specialty.

Consistent with the existing studies our survey also shows a high level of overall career satisfaction among human genetics residents in Germany. The largest survey in Germany concerning career satisfaction of medical residents has been conducted by van den Bussche et al., evaluating longitudinal data of initially over 1,000 German medical residents. In their study gender and parental status significantly influenced career satisfaction, with female residents with children showing a significantly lower career satisfaction than their male colleagues with or without children, a finding consistent with other studies measuring career satisfaction among residents [1], [11], [12]. In our survey, women with children had lower scores with regard to achieving their professional goals and to their perceived progress in their professional life compared to women without children. In contrast, no significant differences were found between men with and men without children. This may be explained by the fact that women still carry a main part of care work. Apart from this, neither parental status, nor gender, nor working part time had a significant effect on career satisfaction. In the German health care system specialist care is provided in both the inpatient and outpatient sectors. For human genetics this means that specialist training can be completed in academic institutions belonging to university hospitals or private practice/medical care centres that offer medical care within the framework of statutory health insurance accredited medical care. In our cohort 65 % of residents received their specialist training at an academic institution and 35 % in private practice/medical care centres. The site of the specialist training had no influence on career satisfaction. It can be speculated that most institutions of our cohort try to install working and career conditions which meet the above-mentioned needs of residents regarding gender equality and reconciliation of family and career, a fact that should be brought into sharper focus when recruiting young residents.

However, the fact that there is a significant difference in research activities between male and female residents, even if this does not seem to have an impact on career satisfaction, should warrant attention. The unequal distribution could not be explained by an unequal distribution of male and female residents with regard to the site of specialist training, assuming that research mainly takes place at academic institutions. In academic institutions more males than females participate in research, as well as more male residents with children than female residents with children. Interestingly, at academic institutions, 80 % of women and 77 % of men have doctorates, so the starting conditions for research are the same, so the women are lost somewhere down the line. Since research activities are a prerequisite for further career progression, this unequal distribution can lead to an unequal recruitment of men and women for leadership positions further down the line. The institutions should investigate potential structural reasons that hamper female residents’ research activities and promote research activities of female residents e. g. by the implementation of programmes for the support of female researchers, mentoring programmes, adapted working time models or networking programmes.

Consistent with previous studies [1], in our model the highest influence on career satisfaction came from the occupational self-efficacy expectations, meaning confidence in the own professional capability. There is a complex relationship between occupational self-efficacy expectations, perceived workload, perceived stress and burnout prevalence, with residents with lower occupational self-efficacy expectations exhibiting higher levels of stress [13]. Overall burnout syndrome prevalence among human residents has been shown to be around 35.7 % [14]. Studies generally show that burnout is associated with an increased workload and that a higher career satisfaction is consistently associated with less burnout [15], [16], [17]. Lower occupational self-efficacy expectations and burnout influence specialty choice already at the stage of medical students [18]. In this context it seems positive that the perceived subjective workload was generally moderate in our cohort, with lowest item scores for the subjective frustration. No correlation between career satisfaction and workload could be observed. However, it also shows that the foundations for a high level of occupational self-efficacy expectations must already be laid in medical school by teaching skills for the successful practice of medicine and by establishing prevention strategies for unrealistic occupational self-efficacy expectations and burnout. In the years of residency, it is up to the doctor and the institution responsible for the specialty training to ensure that there is an appropriate balance between the resident’s existing skills and the transfer of responsibility.

In recent years, it has been increasingly recognized that the individual teacher has a significant influence on learning success and the acquisition of competences [19]. As shown by Buxel, the satisfaction with the specialty training situation, working atmosphere, workload and leadership style greatly influence general job satisfaction [20]. Overall appreciation of leadership, guidance of residents during training, specific instructions and communication skills positively influence job satisfaction, highlighting the impact of the supervisors’ behaviour [21]. In short, it depends on the teacher. This fact has been recognized in the new regulations for specialty training issued by the state medical associations of Germany in 2020 linking the entitlement for specialty training with participation in “train the trainer” courses issued by the medical associations. The importance of evaluating the quality of the specialist training and the doctors entitled to specialty training has long been recognized and is an existing standard in countries like the USA [5]. Especially in smaller institutions, this is rarely carried out despite the existence of validated measurement instruments such as those of Prien et al. because anonymity is not given in the survey and negative effects on the relationship between the doctor entitled to the specialty training and the resident are feared [5]. However, also in line with previous studies [1], our survey shows a significant correlation between the excellence of the doctor entitled to the specialist training and residents’ career satisfaction. The residency institutions and the doctors entitled to the specialist training must be aware of the responsibility and take measures to constantly ensure a permanent good quality of specialist training.

Residents’ assessment of their own professional prospects and the prospects of the subject are consistently positive. However, the results of the survey show that the human genetics community in general and their respective professional societies have two tasks to fulfil: First human genetics as a specialty must be firmly anchored in the consciousness of other medical colleagues and the general population. Secondly, at the political level, conditions must be created to give residents a long-term perspective in the subject. The existing requirement planning guideline not only stands in the way of adequate care; it also leads to the absurd situation that, despite increasing demand for genetic services, not all residents may be able to obtain the authorization to treat statutory health insurance patients by the Association of Statutory Health Insurance Physicians (Kassenärztliche Vereinigung) after passing the specialist examination and could thus be forced to migrate to other disciplines or professions.

## Conclusion

Career satisfaction of German human genetics residents is generally high and mainly influenced by life satisfaction, occupational self-efficacy expectations and quality of the specialist training. In contrast to other specialties career satisfaction seems to be independent from gender or parental status even though male residents were significantly more often involved in research activities. In order to keep human genetics residents in the specialty, measures that enable balanced professional and care work as well as continuous improvement of specialist education, e. g. through the implementation of structured curricula and continuing education of the doctors entitled to specialist training, are of great importance.
